# Cytokine Profiles at Birth Predict Malaria Severity during Infancy

**DOI:** 10.1371/journal.pone.0077214

**Published:** 2013-10-10

**Authors:** Edward Kabyemela, Bronner P. Gonçalves, D. Rebecca Prevots, Robert Morrison, Whitney Harrington, Moses Gwamaka, Jonathan D. Kurtis, Michal Fried, Patrick E. Duffy

**Affiliations:** 1 MOMS Project, Seattle Biomedical Research Institute, Seattle, Washington, United States of America, and Muheza Designated District Hospital, Muheza, Tanzania; 2 Laboratory of Malaria Immunology and Vaccinology, National Institute of Allergy and Infectious Diseases, NIH, Rockville, Maryland, United States of America; 3 Laboratory of Clinical Infectious Diseases – Epidemiology Unit, National Institute of Allergy and Infectious Diseases, NIH, Bethesda, Maryland, United States of America; 4 Seattle Children's Hospital and Department of Pediatrics, University of Washington School of Medicine, Seattle, Washington, United States of America; 5 Center for International Health Research, Rhode Island Hospital, Providence, Rhode Island, United States of America; 6 Department of Pathology and Laboratory Medicine, Brown University Medical School, Providence, Rhode Island, United States of America; Shoklo Malaria Research Unit Mahidol University and University of Oxford, Thailand

## Abstract

**Background:**

Severe malaria risk varies between individuals, and most of this variation remains unexplained. Here, we examined the hypothesis that cytokine profiles at birth reflect inter-individual differences that persist and influence malaria parasite density and disease severity throughout early childhood.

**Methods and Findings:**

Cytokine levels (TNF-α, IFN-γ, IL-1β, IL-4, IL-5, IL-6 and IL-10) were measured at birth (cord blood; N=783) and during subsequent routine follow-up visits (peripheral blood) for children enrolled between 2002 and 2006 into a birth cohort in Muheza, Tanzania. Children underwent blood smear and clinical assessments every 2-4 weeks, and at the time of any illness. Cord blood levels of all cytokines were positively correlated with each other (Spearman’s rank correlation). Cord levels of IL-1β and TNF-α (but not other cytokines) correlated with levels of the same cytokine measured at routine visits during early life (P < 0.05). Higher cord levels of IL-1β but not TNF-α were associated with lower parasite densities during infancy (P=0.003; Generalized Estimating Equation (GEE) method), with an average ~40% reduction versus children with low cord IL-1β levels, and with decreased risk of severe malaria during follow-up (Cox regression): adjusted hazard ratio (95% CI) 0.60 (0.39-0.92), P = 0.02.

**Conclusion:**

IL-1β levels at birth are related to future IL-1β levels as well as the risk of severe malaria in early life. The effect on severe malaria risk may be due in part to the effect of inflammatory cytokines to control parasite density.

## Introduction

Despite renewed efforts at control and elimination, malaria remains a major cause of morbidity and mortality in Africa, where 174 million clinical cases occur annually, resulting in an estimated 596,000 deaths [1]. Although factors such as sickle cell trait are known to influence malaria severity [2], most of the variation in risk between individuals remains unexplained [3]. Human genetics [4], parasite virulence [5], environmental factors [6] and acquired immunity [7] can all contribute to variations in risk.

During infection, cytokines play a dual role by controlling parasite growth on the one hand while exacerbating pathology on the other. These opposing effects have been attributed to the timing of cytokine expression [8] as well as the balance between inflammatory and anti-inflammatory cytokines [9]. For example, the inflammatory cytokines TNF-α and IFN-γ can mediate parasite inhibition and killing [10,11], but high levels of TNF-α have also been associated with severe malaria syndromes such as cerebral malaria [12,13]. Meanwhile, high levels of the anti-inflammatory cytokine IL-10, or high IL-10/TNF-α ratios, reduce the risk of severe malarial anemia [14,15], despite being associated with reduced parasite clearance in children with uncomplicated malaria [16].

Cytokine levels at birth might reflect inter-individual differences that persist and influence malaria outcomes during childhood. In malaria endemic areas, fetal sensitization to malaria antigens is common: cord blood lymphocytes often respond to stimulation with malaria antigens by proliferating [17,18] and producing type 1 and/or type 2 cytokine responses [19]. Some newborns of infected mothers display a “tolerant” phenotype (i.e. PBMC non-responsive to malaria antigens), and have an increased risk of infection and lower hemoglobin levels during early life [20]. 

To test the hypothesis that *in utero* immune profiles will persist during early childhood and influence malaria outcomes, we measured plasma cytokine levels at birth and analysed their relationship with cytokine levels and the risk of severe *P. falciparum* malaria during early life. We report for the first time that high levels of IL-1β in cord blood persist, and are associated with both improved control of parasite density and with decreased risk of severe malaria during infancy. 

## Materials and Methods

### Study population and clinical procedures

Mothers and newborns were enrolled in a birth cohort study known locally as the Mother-Offspring Malaria Studies (MOMS) Project at Muheza Designated District Hospital, Muheza, Tanzania. Clinical procedures for the MOMS Project have been previously described [21]. Children whose data are reported in this study were enrolled between September 2002 and May 2006, and were followed for up to 4 years. Twins, stillbirths, early neonatal deaths, infants with any evidence of HIV infection (mother seropositive on voluntary testing, infant presented with suggestive signs or symptoms or suffered HIV/AIDS-related death during follow-up) or sickle-cell disease were excluded. Of the 882 children who remained after exclusions, 783 had cord blood cytokines measured and were thus included in this analysis. 

Children were examined and blood smears obtained by finger or heel prick every 2 weeks during infancy and every 4 weeks post-infancy. Peripheral blood was collected into CPD anticoagulant during routine visits at roughly 3 months of age and then at ~6 month age intervals thereafter. Whenever children developed symptoms, they were examined by a study clinician. Children were classified as having severe malaria according to WHO criteria [22]. 

### Ethics

Written informed consent was obtained from mothers prior to enrollment. Protocols for procedures used in this study were approved by the International Clinical Studies Review Committee of the Division of Microbiology and Infectious Diseases at the US National Institutes of Health, and ethical clearance was obtained from the Institutional Review Boards of Seattle BioMed and the National Medical Research Coordinating Committee in Tanzania.

### Laboratory procedures

Cord blood samples were obtained by clamping the cord and cannulating umbilical vessels immediately after delivery. After removal of the umbilical cord and fetal membranes, placental blood samples were obtained by manual compression of the placental tissue in a grinder. Placental and cord blood samples were anticoagulated with EDTA, and stored on ice until processing the same day. 

Malaria parasitemia diagnosis: Parasitemia was defined as identification of any parasites in a Giemsa-stained blood smear by microscopy, after counting at least 200 white blood cells at a magnification of 100x.

Determination of red blood cell disorders: Hemoglobin type (HbAA, HbAS and HbSS) was determined by cellulose acetate paper electrophoresis according to the manufacturer’s instructions (Helena Laboratories, Beaumont, Texas, USA). Genotyping for α-thalassemia was done according to the protocol described by Chong et al [23].

Cytokine assays: Plasma was obtained by centrifugation at 3,000 * *g* for 3 minutes and was stored frozen at -70°C until it was thawed on the day that cytokine assays were performed. Each plasma sample was analysed using a multiplex, bead-based platform (BioPlex; Bio-Rad, Irvine, CA) and custom-made assay kits as previously described [24]. For each plasma sample, all analytes were assayed in a single day, thus eliminating freeze-thaw cycles. All pipetting and sample identification were performed with a bar code-enabled, high-speed pipetting robot (Megaflex; Tecan, Research Triangle Park, NC). The detection limits for the different analytes were as follows: TNF-α, 0.10 pg/ml; IFN-γ, 0.04 pg/ml; IL-1β, 0.01 pg/ml; IL-4, 0.3 pg/ml; IL-5, 0.02 pg/ml; IL-6, 1.45 pg/ml; IL-10, 0.02 pg/ml. Cytokine levels were adjusted to account for dilution in anticoagulant at the time of sample collection. 

### Statistical analysis

Non-parametric tests (Mann-Whitney and Kruskal Wallis tests) were used to compare cord blood cytokine levels by baseline variables (gender, parity, placental malaria status, birth season, sickle cell and alpha-thalassemia genotypes). Malaria transmission season was defined as high between May and October, based on the peak incidence of parasitemia observed in our cohort. Correlations between cytokines at birth and at routine uninfected healthy visits were evaluated by Spearman’s rank correlation coefficient. Bonferroni correction was used in the pairwise correlations between cytokines at birth. 

Cox regression models were fitted to evaluate the relationship between cord blood cytokine levels and the time to first episode of severe malaria. Schoenfeld residuals were used to test the proportional hazards assumption. Kaplan-Meier curves were used to display severe malaria rates in children with high versus low levels of cord cytokines IL-1β and TNF-α. To account for the correlation between visits of the same child, the association between cord blood cytokines and parasite density (log-transformed) was assessed by Generalized Estimating Equation (GEE) method. In this analysis, each positive blood smear was included as an observation. Exchangeable correlation structure and identity link function were used; and robust standard errors were estimated. Cytokine levels were included in Cox and GEE models as binary variables, determined by the median: values above the median were considered high and values below the median, low. Median values (in pg/ml) for cord blood cytokines were: TNF-α, 120.8; IL-1β, 6.0; IL-5, 2.6; IL-6, 7.0; IL-10, 3.5. Models were adjusted for sickle cell trait status, alpha-thalassemia, transmission season, bed net use, and village of residence. Since parity and placental malaria interact to influence malaria infection and clinical malaria risk [21,25], these variables were included with interaction term.

**Figure 1:**
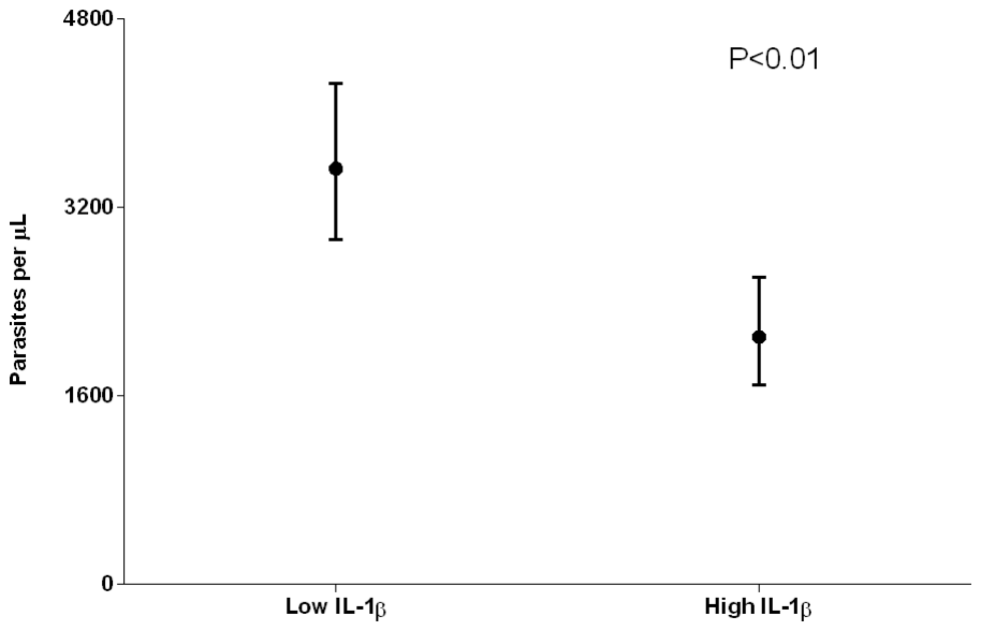
Average parasite densities in children with high and low cord IL-1β levels. Only infections occurring in the first year of life were included in this analysis, since the association between levels of this cytokine at birth and subsequent parasite levels was only present during infancy (GEE model). (N=504, children with at least one infection during infancy) Concentrations of parasites per μL were estimated by assuming 8000 leukocytes/μL of blood. Children were defined as having high cord IL-1β levels if their IL-1β levels at birth were higher than the median in the study population (6 pg/ml); if IL-1β levels at birth were lower than the median value, these levels were considered low.

Average parasite densities during infections in infants, children aged 1 year or less, were estimated for children with high and low cord IL-1β levels: geometric mean parasite density during visits with infection was calculated for each child; the geometric mean of these values was then estimated for groups with different IL-1β levels at birth (Figure 1).

Data analyses were conducted using STATA version 11.1 (Stata Corporation, College Station, Texas, United States)

## Results

### Description of the study cohort

105 (13.4%) newborns were delivered by mothers with placental malaria (PM+), and this was most common in first and second time mothers (19.3%, 18.5% and 7.6% in primiparae, secundiparae and multiparae, respectively; P < 0.001) (Table **1**). Mean birth weight was lower in offspring of PM+ versus PM- women in all parity groups (P < 0.05 for all groups) [26]. 61.5% of children used bed nets. Sickle cell trait was frequent in this population (16.6%).

**Table 1:** Demographic characteristics of the cohort.

	**Number (%)**
**Parity**	
*Primiparae*	218 (27.8)
*Secundiparae*	184 (23.5)
*Multiparae*	381 (48.7)
**Gender**	
*Female*	379 (48.4)
*Male*	404 (51.6)
**Placental Malaria (PM) status**	
*PM+*	105 (13.4)
*PM-*	678 (86.6)
**Birth season**	
*High*	367 (46.9)
*Low*	416 (53.1)
**Residence**	
*Bwembwera*	132 (16.9)
*Magilla*	130 (16.6)
*Mkanyageni*	169 (21.6)
*Muheza township*	352 (45.0)
	**Median (Q1-Q3)**
**Follow-up duration (in years)**	2.15 (1.22-2.98)
	**Mean (SD)**
**Number of visits per child**	44.6 (19.6)

### Cord blood cytokine levels vary in relation to *in utero* factors

Cord blood levels of some cytokines differed according to malaria transmission season, placental malaria status and maternal parity (Table **2**). Cord blood IFN-γ was more often detected during high versus low malaria transmission season among primi- (P=0.06) and multiparae (P<0.001), but not secundiparae (P=0.58). Placental malaria and birth during high transmission season were associated with higher cord blood levels of IL-10 (P=0.04 and P=0.05, respectively), a cytokine that is also elevated in maternal samples during episodes of inflammatory placental malaria [27,28]. Cord blood levels of IL-10 were also significantly higher in primiparae than other groups (P=0.003). Cord IL-4 was more frequently detected in children born during high transmission season (P=0.002). Sickle cell trait and alpha-thalassemia in the children did not influence levels of cord blood cytokines.

**Table 2:** Cord cytokine levels stratified by parity, transmission season and placental malaria status (Median [Q1-Q3]).

	**Parity**					**Transmission Season**				**Placental Malaria**		
	*Primiparae*	*Secundiparae*	*Multiparae*	*p-value*		*High*	*Low*	*p-value*		*PM +*	*PM -*	*p-value*
**TNF-α**	118.7 (70.5 - 180.9)	132.7 (72.2 - 201.9)	116.5 (68.1 - 173.8)	0.15		122.8 (72.1–192.7)	116.9 (64.5 - 173.5)	0.31		128.9 (79.7 - 191)	119.6 (68.2 - 180.7)	0.29
**IL-1β**	5.8 (2.9 - 10.7)	6.4 (3.0 - 11.8)	5.9 (3 - 11.9)	0.8		5.8 (3 - 11.1)	6.3 (2.9 - 11.9)	0.84		5.8 (3.1 - 9.9)	6.1 (3 - 11.9)	0.5
IL-4 *	12.4	9.8	10.5	0.67		14.4	7.7	0.002		10.5	10.9	0.89
**IL-5**	2.4 (0.7 - 4.9)	2.7 (1 - 5.2)	2.7 (1.1 - 5.3)	0.35		2.9 (0.7 - 5.4)	2.4 (1 - 5.2)	0.45		2.5 (0.4 - 6)	2.6 (1 - 5.2)	0.39
**IL-6**	7.3 (2.3 - 17.5)	5.6 (1.3 - 15.3)	7.7 (2.4 -20.9)	0.13		6.9 (2.3 -22.3)	7 (1.9 - 16.2)	0.44		5.6 (1.2 - 12.8)	7.1 ( 2.3 - 19.1)	0.14
**IL-10**	4 (2.1 - 6.9)	2.9 (1.3 - 5.5)	3.3 (1.3 - 5.8)	0.003		3.6 (1.6 - 6.5)	3.2 (1.5 - 5.3)	0.05		3.9 (1.9 - 6.9)	3.3 (1.5 - 5.9)	0.04
IFN-γ *	22.9	21.7	18.6	0.41		25.6	16.1	0.001		20.5	20.9	0.91

Cytokine levels are presented as pg/ml.

* Percentage with detectable cytokine

### Pro- and anti-inflammatory cytokines in cord blood are positively correlated

Pro-inflammatory and anti-inflammatory cytokines can be counter-regulatory. We analysed pairwise relationships between and within these two groups of cytokines at birth (Table **3**). The pro-inflammatory cytokines TNF-α and IL-1β were highly correlated and both were correlated to IL-4 and IL-10 levels, which suggests that cytokines in cord blood were most likely influenced by a common process (eg, inflammation) rather than factors that alter expression of specific cytokines (eg, single nucleotide polymorphisms). 

**Table 3:** Correlation between cord cytokine levels (Spearman’s rank correlation).

	**TNF-α**	**IL-1β**	**IL-4**	**IL-5**	**IL-6**	**IL-10**	**IFN-γ**
**TNF-α**							
**IL-1β**	0.64						
**IL-4**	0.22	0.23					
**IL-5**	0.4	0.34	0.17				
**IL-6**	0.14	0.37	0.13	0.15			
**IL-10**	0.32	0.34	0.23	0.27	0.45		
**IFN-γ**	0.35	0.3	0.34	0.25	0.16	0.34	

All correlations had Bonferroni-adjusted p-values<0.001, except correlations between IL-6 and TNF-α (P=0.002) and between IL-6 and IL-4 (P=0.003)

### IL-1β and TNF-α levels at birth correlate with levels during early childhood

We examined the relationship between cord levels of cytokines at birth, and levels measured in peripheral blood of these children during follow-up (Table **4**). We included only measurements made at visits when the child was healthy and aparasitemic (N = 1,359 visits). IL-4 and IFN-γ levels were often undetectable, and therefore we used logistic regression to analyse these cytokines as binary variables (detectable vs. undetectable). Cord levels of TNF-α and IL-1β correlated significantly to levels measured later in life: TNF-α at birth correlated to levels measured throughout childhood, while IL-1β correlated to levels measured during the first year of life but not thereafter. Cord levels of other cytokines measured in this study did not correlate with peripheral blood levels throughout infancy or early childhood.

**Table 4:** Correlation between birth levels and early childhood levels of cytokines.

**Age (in weeks)**	**TNF-α**	**IL-1β**	**IL-5**	**Il-6**	**IL-10**	**Number of Children**
*< 12*	0.27*	0.16*	0.1	0.01	0	162
*12-24*	0.18*	0.14*	0.06	0.07	0.04	287
*24-48*	0.30*	0.21*	0.05	-0.01	0.04	229
*48-76*	0.14*	0.07	0.05	0.05	0.05	225
*76-100*	0.18*	-0.04	-0.09	0.07	0.08	182
*100-124*	0.23*	0.07	0.1	-0.07	-0.01	146
*124-148*	0.28*	0.02	0.01	0.05	-0.02	88

Correlation coefficients (Spearman’s rank correlation) at different age intervals are presented. IL-4 or IFN-γ were only detected in a minority of samples, and were therefore analyzed by logistic regression to assess whether cytokine positivity at birth predicted cytokine positivity during childhood. Detectable cord levels of IL-4 were associated with detectable IL-4 in samples collected before 12 weeks of age (odds ratio 2.46 95%CI [0.93 - 6.52], P=0.07). Detectable IFN-γ levels at birth were associated with IFN- γ detection in the first 12 weeks of life (odds ratio 2.26 95%CI [1.08 - 4.72], P=0.03), and with IFN- γ negativity between weeks 124 and 148 (odds ratio 0.20 95%CI [0.06 - 0.68], P=0.01).

* P<0.05

### High IL-1β levels at birth predict reduced parasite densities and severe malaria risk in infancy

During a malaria infection, pro-inflammatory cytokines are rapidly released from innate and adaptive immune cells, and may contribute to control of parasite density [29]. We analysed the relationship between cord levels of the cytokines IL-1β and TNF-α, and parasite densities during subsequent infections. High levels of IL-1β in cord blood were related to lower parasite levels during infections in infants (P=0.003, GEE model [Table 5]), equal to ~40% reduction in average parasite density (geometric mean parasite density in children with high cord IL-1β 2100 95% CI [1692 - 2607] versus children with low cord IL-1β 3528 95%CI [2927 - 4253] parasites per μL; Figure 1). Cord TNF-α levels did not predict parasite densities during subsequent infections.

**Table 5:** Cox model on time to first severe malaria episode and GEE model on parasite density.

	*Severe Malaria*	*Parasite density*
**Cytokine**	Cox Model*	***GEE model* (*< 1st year*)^¤^**
IL-1β	0.60 (0.39-0.92) P=0.02	-0.16 (-0.26 -0.05) P=0.003
*TNF-*α	0.68 (0.45-1.03) P=0.07	-0.09 (-0.20 - 0.01) P=0.09

For Cox regression results, hazard ratios adjusted for factors that might influence severe malaria risk (sickle cell trait status, alpha-thalassemia, transmission season, parity vs. placental malaria, bed net use and village of residence) are shown. Regression coefficients are presented for GEE models that assess the influence of IL-1β or TNF-α on parasite density

* Hazard ratio

¤ Regression coefficients

**Figure 2:**
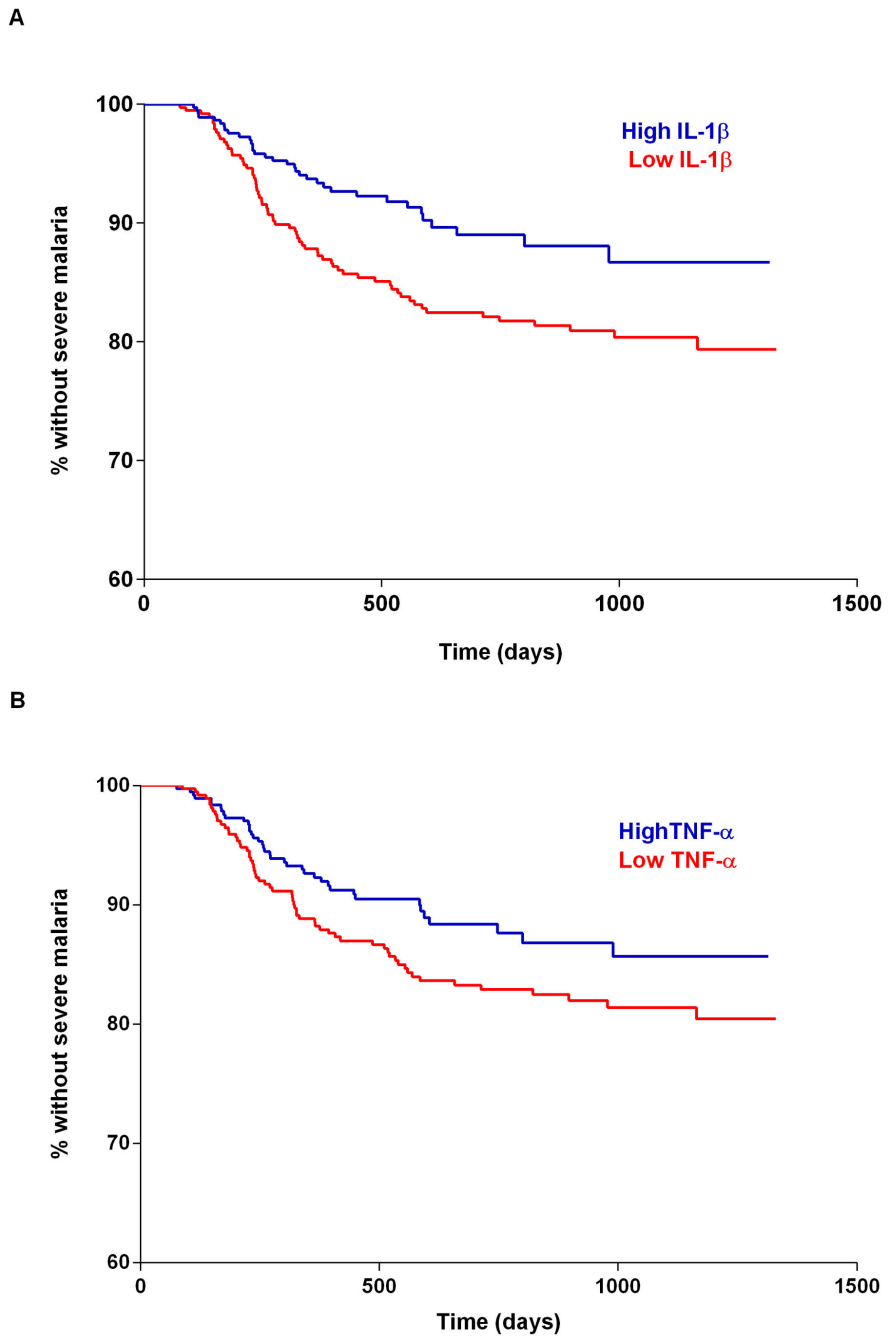
Kaplan-Meier curves for the risk of severe malaria. (a) High cord levels of IL-1β were associated with longer time to first severe malaria episode (P=0.008, log-rank test); (b) High levels of TNF-α at birth were marginally associated with a longer time to first severe malaria episode (P=0.08, log-rank test). High levels of cord IL-1β and TNF-α were defined based on median values (TNF-α 120.8 pg/ml; IL-1β 6 pg/ml).

Finally, we analysed the influence of cord blood TNF-α and IL-1β levels on severe malaria risk. Kaplan-Meier curves (Figure 2) indicate that children with high cord levels of IL-1β (but not TNF-α) are protected against severe malaria. In Cox regression analyses, high levels of IL-1β at birth decreased the risk of first severe malaria episode (hazard ratio (95% CI, P-value) of 0.60 (0.39 - 0.92, P = 0.02)) (Table 5). In a multivariate Cox model that included all cord blood cytokines as well as other baseline covariates, only IL-1β levels at birth had a significant effect on the time to first severe malaria event, reducing the risk of severe malaria episode by 42% (hazard ratio (95% CI, P-value) 0.58 (0.35 - 0.97 , P=0.04)).

## Discussion

In this study, we investigated whether cord blood cytokines of children living in a malaria endemic area are related to cytokine levels during early life and whether they predict severe malaria risk. Our results show for the first time that higher levels of the pro-inflammatory cytokine IL-1β at birth are significantly associated with improved control of parasite density and with decreased risk of severe malaria during early life. These findings support the idea that *in utero* sensitization or constitutive expression levels of cytokines, reflected by cord blood cytokine levels, contributes to the risk of severe malaria during childhood. 

Previous studies have shown that cord blood cytokine responses might influence the risk of common childhood diseases. In the US, greater IFN-γ secretion by cord blood mononuclear cells (CBMC) reduces the risk of acute lower respiratory illness in the first year of life [30]. In a prospective birth cohort study in Kenya, children of malaria-infected women whose CBMC did not produce cytokines (IFN-γ, IL-2, IL-13, and/or IL-5) in response to blood stage malaria antigens were at increased risk of infection and had lower hemoglobin levels during childhood [20]. The effect of specific cytokines or immune responses on malaria severity has not previously been assessed.

In our birth cohort, infants with high levels of TNF-α and IL-1β at birth persist in this pattern during infancy, and these intrinsically higher levels might act early during an infection to control parasite density. TNF-α is a major effector cytokine and is implicated in both protection and pathogenicity during malaria infection. Several studies have observed that plasma levels of TNF–α are significantly higher in infected humans presenting with severe malaria [12,13]. In our study, high cord levels of TNF-α were associated with decreased risk of severe malaria, although this association was only marginally significant. The protective role of TNF-α is suggested by animal studies in which malaria-resistant C57BL/6 mice had higher levels of TNF-α mRNA in the spleen and liver during the early phase of infection, which enhanced clearance of infection [31]. In humans, TNF-α production during the acute phase of malaria similarly predicts a more rapid clinical and parasitological cure [32,33]. In a prospective study from Papua New Guinea, children with increased TNF-α levels during a *P. falciparum* infection were at lower risk of subsequent *P. falciparum* clinical episodes [34], which is consistent with more rapid clearance of parasites. In Gabon, children with a history of severe malaria had fewer T cells producing TNF-α in response to parasite antigen than children with a history of only mild malaria [35].

IL-1 acts synergistically with TNF-α to enhance NO and IFN-γ production in murine models of malaria [36]. PBMC IFN-γ production in response to malaria antigens has been associated with protection against reinfection in children with mild malaria [37], and NO has a direct parasite killing effect [38]. IL-1 also inhibits the intra-hepatocytic development of the rodent malaria parasite *P. yoelii*, an effect partly mediated by IL-6 secretion [39], and controls blood stage parasitemia in mice infected with *P. berghei* [40]. Our finding that high levels of cord blood IL-1β reduce the risk of severe malaria is also consistent with a study showing that IL-1β promoter haplotype -31C/-511A is associated with decreased production of IL-1 and increased risk of severe malarial anaemia in Kenyan children [41]. In our cohort, the protection associated with cord IL-1β against both parasite density and SM during infancy (reductions of ~40% and 40%, respectively) was similar in degree to the independent effects of HbAS throughout early childhood (reductions of 42% and 43%). Future studies should assess the contribution of polymorphisms affecting the inflammasome, a multi-protein complex responsible for processing and secretion of IL-1β: mutations that enhance inflammasome activity might result in increased levels of IL-1β at birth and afterwards.

Malaria infection during pregnancy modifies the risk of malaria infection and disease for the offspring [25,42]. In Tanzania, we previously observed that offspring of infected multiparae but not primiparae have increased risk of malaria infection [21], and more recently that these offspring have an increased risk of severe malaria (manuscript submitted). In Gabon, Schwarz et al found that the risk of clinical malaria is higher in children born to multigravidae with placental malaria [25]. We speculated that the effect of pregnancy malaria on malaria outcomes in their offspring might be mediated by altered cytokine responses in offspring that would be evident at birth. However our analyses do not identify a relationship of placental malaria at delivery to cord levels of IL-1β. Further research is warranted to identify the mechanisms by which maternal malaria modifies the risk of childhood malaria in offspring. 

Future studies should also determine the mechanisms that control or modify cord blood cytokine profiles. Other maternal infections endemic in the study area such as lymphatic filariasis could be modifying cord blood cytokine production, but were not studied in this cohort. Alternatively, children may have intrinsic differences in cytokine expression, owing to, for example, genetic polymorphisms or *in utero* imprinting, and these differences could influence parasite density and severe malaria risk during early life. We have found that all cytokines tested are positively correlated at birth, suggesting that cytokine levels in cord blood might have been influenced by a common mechanism (such as inflammation) more so than by individual genetic polymorphisms.

Severe malaria represents a heterogeneous group of clinical presentations, including severe anemia, cerebral malaria, and respiratory distress, among others. These different syndromes might be associated with specific immune responses: for example, children with severe anemia have lower IL-10 levels compared to children with cerebral malaria [14,43]. Future studies with sufficient sample sizes should assess whether elevated IL-1β levels at birth reduce risk of all severe malaria symptoms equally or only of specific syndromes, and also whether a threshold level of IL-1β is required for the protective effect. 

In conclusion, IL-1β levels in cord blood predict IL-1β levels, parasite density, and severe malaria risk throughout infancy. Therefore, factors that influence the cytokine profile or pro-inflammatory bias of the unborn child may have a significant impact on the outcome of malaria infections throughout early life. Placental malaria also increases parasite density and severe malaria risk in offspring, but it does not influence cord cytokine levels and therefore acts independently of the cord cytokine effect. Further study is needed to identify the factors that influence the fetal cytokine profile, as well as interventions that might target these factors to improve malaria outcomes during early childhood. 
